# Idiopathic pulmonary fibrosis in small cell lung cancer as a predictive factor for poor clinical outcome and risk of its exacerbation

**DOI:** 10.1371/journal.pone.0221718

**Published:** 2019-08-23

**Authors:** Nobuyuki Koyama, Yuki Iwai, Yoshiaki Nagai, Kazutetsu Aoshiba, Hiroyuki Nakamura

**Affiliations:** 1 Department of Clinical Oncology, Tokyo Medical University Ibaraki Medical Center, Ibaraki, Japan; 2 Department of Respiratory Medicine, Tokyo Medical University Ibaraki Medical Center, Ibaraki, Japan; 3 Department of Respiratory Medicine, Jichi Medical University, Tochigi, Japan; American Society for Investigative Pathology, UNITED STATES

## Abstract

**Objective:**

Lung cancer frequently co-exists with idiopathic interstitial pneumonia (IIP), which can be subdivided into idiopathic pulmonary fibrosis (IPF) and IIP other than IPF (other IIP). Although chemotherapy in small cell lung cancer (SCLC) patients with IIP may result in the exacerbation of IIP, these patients commonly receive chemotherapy. This study aimed to assess the risks and benefits of chemotherapy in SCLC patients with IIP.

**Methods:**

We retrospectively analyzed the medical records of 122 patients with SCLC who received chemotherapy. Patients with secondary interstitial lung disease (ILD) of known etiology were excluded. Eligible patients were divided into two groups: SCLC with and without IIP. The former group was subdivided into those with IPF and other IIP.

**Results:**

Of the 47 (39.2%) SCLC patients with IIP, 20 had IPF and 27 had other IIP. The frequency of chemotherapy-induced ILD development or IIP exacerbation was higher in patients with IPF (40.0%) than in those with other IIP (3.7%) and non-IIP (1.4%). Logistic regression analysis demonstrated that ILD development or IIP exacerbation was independently associated with IPF (*P* = 0.007). Time to treatment failure (*P* < 0.001) and overall survival (*P* = 0.001) were different among the groups., Cox proportional hazard model revealed that IPF was independently associated with time to treatment failure (*P* = 0,017) and overall survival (*P* = 0.006). Other IIP had no impact on time to treatment failure or overall survival. Development of ILD or exacerbation of IIP independently reduced time to treatment failure and overall survival.

**Conclusions:**

Comorbid IPF can be an independent, negative prognostic indicator and at high risk of ILD development or IIP exacerbation in SCLC patients. Early diagnosis and intervention for chemotherapy-induced IIP exacerbation will be beneficial for SCLC patients with IPF, who need close monitoring for its onset.

## Introduction

Interstitial lung diseases (ILD) are a heterogeneous group of diffuse parenchymal lung diseases with a variety of etiologies, which include genetic predisposition and environmental factors. According to the American Thoracic Society ATS)/European Respiratory Society (ERS)/Japanese Respiratory Society (JRS), idiopathic interstitial pneumonia (IIP) of unknown etiology is a form of ILD, and it is further subdivided into multiple disease categories [1]. Out of these categories, usual interstitial pneumonia (UIP)/idiopathic pulmonary fibrosis (IPF) accounts for 80–90% of IIP cases, and patients with IPF has a poor prognosis, with a median survival time of 3–5 years [2–5]. The poor prognosis is due to the lack of therapeutic options, drug resistance, and the frequency of acute exacerbation, compared with other types of IIP [1].

While the etiology of IIP remains unknown, scientific evidence suggests a common pathogenic mechanism between IIP and lung cancer [6, 7]. This may explain the high rate of comorbidity, and the fact that lung cancer occurs in 4.4–48% of patients with IIP [8–10]. Thus, IIP is considered as an independent risk factor for lung cancer [3]. IPF has also been linked to a higher incidence of lung cancer compared with IIP other than IPF (other IIP) [11]. However, anti-cancer treatments, such as chemotherapy, thoracic radiation, and surgical resection, may result in an exacerbation of IIP, which raises the question of whether patients with the comorbidity of lung cancer and IIP should be treated with these therapeutic modalities.

Lung cancer is the leading cause of cancer-related deaths worldwide. Small cell lung cancer (SCLC), which accounts for 10–20% of lung cancer cases, is characterized by tumor invasiveness, rapid progression, and metastatic potential [12–14]. The prognosis for patients with SCLC is poor despite its high chemosensitivity [15]. Cigarette smoking is a common causative factor for SCLC and IIP. Coincidentally, these two conditions are usually comorbid [16, 17]. When there is a comorbidity of SCLC with IIP, clinicians usually provide aggressive anti-cancer treatment because the life expectancy of untreated SCLC is 3.7 months, which is worse than that of non-small cell lung cancer [18]. Previous reports of patients with SCLC and ILD including IIP revealed that comorbid ILD was a negative prognostic factor for SCLC, but that patients with both conditions benefited from chemotherapy [19–21]. Kenmotsu et al. reported that patients with lung cancer and comorbid ILD with a UIP pattern identified on computed tomography (CT) had a higher incidence of chemotherapy-induced exacerbation of ILD and a shorter overall survival, compared to patients with a non-UIP pattern of ILD [22]. However, how the comorbidity of IIP as the predominant ILD subtype has an impact on the clinical course of SCLC remains unclear. Particularly, whether in patients with SCLC, the prognosis of comorbid IPF is different from that of other IIP subtypes is crucial for therapeutic decision-making for SCLC.

In this study, we retrospectively compared the clinical information and outcomes between these three groups of patients with SCLC: (a) patients without pre-existing IIP, (b) patients with IPF, and (c) other IIP patients. The characteristic findings on the impact of IPF on the clinical outcome of patients with SCLC may offer valuable insights into the therapeutic strategies for the management of SCLC with IIP.

## Methods

### Patients

We reviewed the medical records of 122 patients with SCLC who underwent chemotherapy at Jichi Medical University Saitama Medical Center from 2008 to 2016 and at Tokyo Medical University Hachioji Medical Center from 2016 to 2017. The institutional review boards of each institute approved this research (# 15–12 and H-196). The retrospective data were analyzed anonymously and patients were given the opportunity to opt out of this study. Thus, a waiver of written or oral informed consent was granted from the institutional review board. The exclusion criteria were patients with secondary ILDs of known etiologies such as sarcoidosis, pneumoconiosis, asbestos-associated lung disease, hypersensitive pneumonitis, and auto-immune disease. Eligible patients were divided into two groups according to the presence or absence of comorbid IIP. Based on the ATS/ERS/JRS statement, patients with IIP were further subdivided into two groups: (a) patients with IPF and (b) those with other IIP. All the patients received cytotoxic chemotherapy. The attending physicians chose chemotherapeutic regimens based on age, performance status, clinical stage, the comorbidity, and patients’ wishes, and decided on dose reduction or treatment cessation depending on the onset of adverse events and disease progression. Chemotherapy was initiated in 16 patients after the diagnosis of SCLC was made by surgical resection.

### Study assessment

After obtaining approval of the institutional review boards, the medical records and CT images were reviewed. The tumor response to chemotherapy was evaluated according to the Response Evaluation Criteria in Solid Tumors (RECIST) version 1.1 [23]. The maximal effect was defined as complete response (CR), partial response (PR), stable disease (SD), or progressive disease (PD). The objective response was defined as CR and PR, and disease control was defined as CR, PR, and SD. Therapeutic efficacy was assessed by the objective response rate and the disease control rate, and therapeutic benefit was evaluated based on the time from the initiation of first-line chemotherapy to the confirmation of treatment failure (time to treatment failure), and the death of the patient (overall survival). Adverse events associated with chemotherapy were confirmed by the medical records review and were evaluated based on the Common Terminology Criteria for Adverse Events (CTCAE) v4.0.

### Statistical analysis

Chi-square and Mann–Whitney U tests were used to evaluate the differences in clinicopathological characteristics between the patient groups. We calculated the time to treatment failure and overall survival using the Kaplan–Meier method, and the survival curves were compared using the log-rank test as univariate analysis. Potential confounding factors for survival time and ILD development/exacerbation were assessed with the Cox proportional hazard model and multivariate logistic regression analysis. A *p* value < 0.05 was considered significant. All statistical analyses were performed using SPSS software version 25 (IBM, Armonk, NY, USA).

## Results

### Patient characteristics

**Fig 1:**
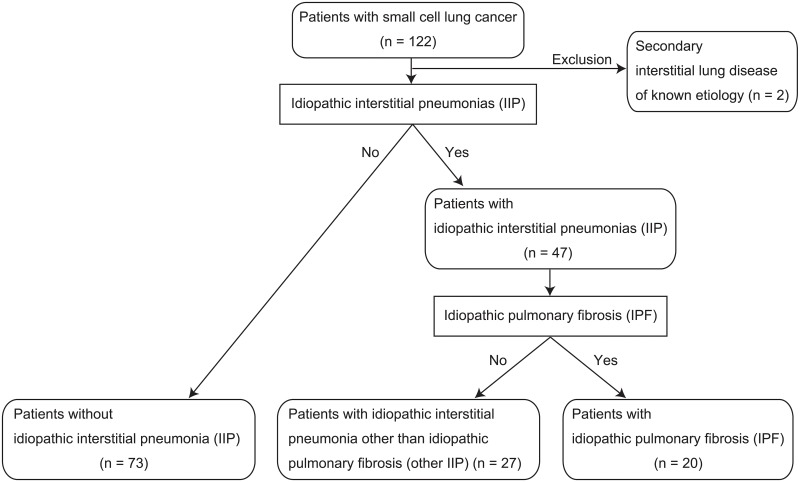
Flow diagram for classification of patient groups in the study. Patients with small cell lung cancer were divided into two groups: patients with idiopathic interstitial pneumonia (IIP) and those without IIP. The group with IIP was subdivided into those with idiopathic pulmonary fibrosis (IPF) and IIP other than IPF.

Of the 120 patients enrolled in this study, 47 patients had IIP. They were further sub-divided into 20 patients with IPF and 27 patients with other IIP (Fig 1). Patients with IIP were older than those without IIP (Table 1). Patients in the IPF and other IIP groups were also older than those without IIP, respectively, whereas there was no significant age difference between the IPF and other IIP groups. Patients without IIP who had a better performance status more frequently underwent first-line chemotherapeutic regimen of cisplatin and etoposide than those with IIP (*P* = 0.027). Patients without IIP more frequently received thoracic radiation therapy. An imbalance in regimens among the patient groups was mainly due to the physicians’ choice in the retrospective study.

**Table 1:** Patient characteristics.

Characteristics	All (n = 120)	Non-IIP (n = 73)	IIP (n = 47)		*P*-value			
			IPF (n = 20)	Other IIP (n = 27)	IIP vs Non-IIP	IPF vs Non-IIP	Other IIP vs Non-IIP	IPF vs Other IIP
**Age (Median years ± SD)**	70±7.9	68±7.8	72±7.1	73±7.6	0.001 >	0.007	0.006	0.880
70 ≤	61	29	14	18				
70 >	59	44	6	9				
**Sex**					0.715	0.770	0.612	0.615
Male	95	57	15	23				
Female	25	16	5	4				
**ECOG performance status**					0.018	0.259	0.014	0.230
0	31	26	3	2				
1	61	32	13	16				
2	18	9	3	6				
3	8	4	1	3				
4	2	2	0	0				
**Smoking (Average pack-year ± SD)**	57±34.5	56±34.6	55±36.8	62±33.5	0.265	0.580	0.251	0.477
40 ≤	86	22	5	7				
40 >	34	51	15	20				
**Stage**					0.285	0.926	0.103	0.172
Limited stage	48	32	9	7				
Extensive stage	72	41	11	20				
**1st-line treatment**					0.027	0.159	0.058	0.703
Cisplatin/Etoposide	19	17	1	1				
Carboplatin/Etoposide	93	51	18	24				
Cisplatin/Irinotecan	3	2	0	1				
Carboplatin/Irinotecan	4	3	1	0				
Amrubicin	1	0	0	1				
**Treatment cycles**					0.090	0.106	0.240	0.538
1	12	6	5	1				
2	8	2	1	5				
3	11	6	1	4				
4	79	53	12	14				
5	3	2	0	1				
6	7	4	1	2				
**2nd-line treatment**					0.749	0.093	0.343	0.063
+	66	41	7	18				
-	54	32	13	9				
**Thoracic radiation**					0.019	0.767	0.157	0.962
+	29	23	2	4				
-	91	50	18	23				
**Surgical intervention**					0.247	0.286	0.614	0.831
+	17	13	1	3				
-	103	60	19	24				
**ILD development or IIP exacerbation**								
+	10	1	8	1	0.002	0.001 >	0.949	0.006
-	110	72	12	26				

IIP, idiopathic interstitial pneumonia; IPF, idiopathic pulmonary fibrosis; Other IIP, idiopathic interstitial pneumonia other than idiopathic pulmonary fibrosis; SD, standard deviation; ECOG, eastern cooperative oncology group; ILD, interstitial lung disease.

### Development of ILD or exacerbation of IIP

Development of ILD or exacerbation of IIP (ILD development/IIP exacerbation) during chemotherapy was identified in 10 patients with SCLC (8.3%). Half of these events occurred during first-line chemotherapy (Table 2). Therefore, patients with ILD development/IIP exacerbation underwent fewer cycles of chemotherapy, compared with those without ILD development/IIP exacerbation. One patient without IIP exhibited a newly-developed ILD (1.4%), whereas IIP was exacerbated in eight patients with IPF (40.0%), and in one patient with other IIP (3.7%). The exacerbation of IIP occurred primarily in patients with IPF compared with those with other IIP. There was no significant difference in the incidence between patients with other IIP and those without IIP. Five patients with ILD development/IIP exacerbation received thoracic radiation therapy, whereas ILD development/IIP exacerbation occurred during concurrent chemoradiation in two patients: one in the non-IIP group and the other in the IPF group, respectively. All patients who had ILD development/IIP exacerbation were treated with corticosteroids after its diagnosis. Only one of 10 patients with ILD development/IIP exacerbation (10.0%) received second-line treatment, whereas 65 of 110 patients without ILD development/IIP exacerbation (59.1%) did not.

**Table 2:** Characteristics of patients with ILD development or IIP exacerbation.

Characteristics	ILD development or IIP exacerbation (+) (n = 10)	ILD development or IIP exacerbation (-) (n = 110)	*P*-value
**Age (Median years ± SD)**	71±5.8	69±8.0	0.161
70 ≤	8	53	
70 >	2	67	
**Sex**			0.198
Male	10	85	
Female	0	25	
**ECOG Performance status**			0.699
0	2	29	
1	7	54	
2	1	17	
3	0	8	
4	0	2	
**Smoking (Average ack-year ± SD)**	52±13.1	58±35.8	0.726
40 ≤	8	78	
40 >	2	32	
**Stage**			0.736
Limited stage	4	44	
Extensive stage	6	66	
**1st-line treatment**			0.990
Cisplatin/Etoposide	1	18	
Carboplatin/Etoposide	9	84	
Cisplatin/Irinotecan	0	1	
Carboplatin/Irinotecan	0	3	
Amrubicin	0	4	
**Treatment cycles**			0.003
1	5	7	
2	0	8	
3	1	10	
4	4	75	
5	0	3	
6	0	7	
**2nd-line treatment**			0.008
+	1	65	
-	9	45	
**Thoracic radiation**			0.148
+	5	26	
-	5	84	
**Surgical intervention**			0.937
+	1	16	
-	9	94	

ILD, interstitial lung disease; IIP, idiopathic interstitial pneumonias; SD, standard deviation;

ECOG, eastern cooperative oncology group.

There was a large difference in the number of patients in the two groups; 10 patients who had ILD development/exacerbation versus 110 patients who did not (Table 2). However, the multivariate logistic regression analyses showed that ILD development/IIP exacerbation was independently associated with IPF (Table 3).

**Table 3:** Logistic regression analysis of ILD development or IIP exacerbation.

	*P*-value	Odds ratio	95% CI
**IPF**	0.007	101.46	3.55–2904.17
**Other IIP**	0.806	1.66	0.03–97.53
**Age**	0.217	1.10	0.95–1.27
**Smoking history (pack-year)**	0.610	1.01	0.98–1.05
**ECOG performance status**	0.425	0.47	0.07–3.03
**Stage (Limited stage vs Extensive stage)**	0.617	0.46	0.02–9.39
**Therapeutic response (CR, PR, SD, PD)**	0.835	0.80	0.09–6.67
**Treatment courses of 1st-line chemotherapy**	0.033	0.20	0.05–0.88
**Thoracic radiation therapy (Yes or No)**	0.089	16.93	0.65–440.78
**1st-line treatment regimens**	0.767	0.75	0.09–6.16
**Surgical intervention (Yes or No)**	0.608	3.69	0.03–540.99

ILD, interstitial lung disease; CI, confidence interval; IPF, idiopathic pulmonary fibrosis;

Other IIP, idiopathic interstitial pneumonia other than idiopathic pulmonary fibrosis;

ECOG, eastern cooperative oncology group; CR, complete response; PR, partial response;

SD, stable disease; PD, progressive disease.

### Therapeutic response to first-line chemotherapy

Patients with IPF had the lowest objective response rate and disease control rate for first-line chemotherapy; particularly, the objective response rate was 60% only in patients with IPF (Table 4). However, the therapeutic response to first-line chemotherapy in patients without IIP was not significantly different from that in patients with IIP, IPF, or other IIP, despite an imbalance in chemotherapeutic regimens between the patient groups. Furthermore, there were no significant differences in therapeutic responses between patients with IPF and those with other IIP.

**Table 4:** Therapeutic response to first-line chemotherapy.

Patients	Treatment regimen	CR	PR	SD	PD	NE	Objective response rate (%)	Disease control rate (%)	*P*-value	
**All patients (n = 120)**	Total	12	65	14	16	13	72	85		
**No IIP (n = 73)**	Total	10	37	5	10	11	76	84		
									IIP vs No IIP	
**IIP (n = 47)**	Total	2	28	9	6	2	67	87	0.149	
									IPF vs No IIP	IPF vs Other IIP
**IPF (n = 20)**	Total	1	11	5	3	0	60	85	0.156	0.501
									Other IIP vs No IIP	
**Other IIP (n = 27)**	Total	1	17	4	3	2	72	88	0.367	

CR, complete response; PR, partial response; SD, stable disease; NE, not evaluated; IIP, idiopathic interstitial pneumonias;

IPF, idiopathic pulmonary fibrosis; Other IIP, idiopathic interstitial pneumonia other than idiopathic pulmonary fibrosis.

### Time to treatment failure and overall survival

**Fig 2:**
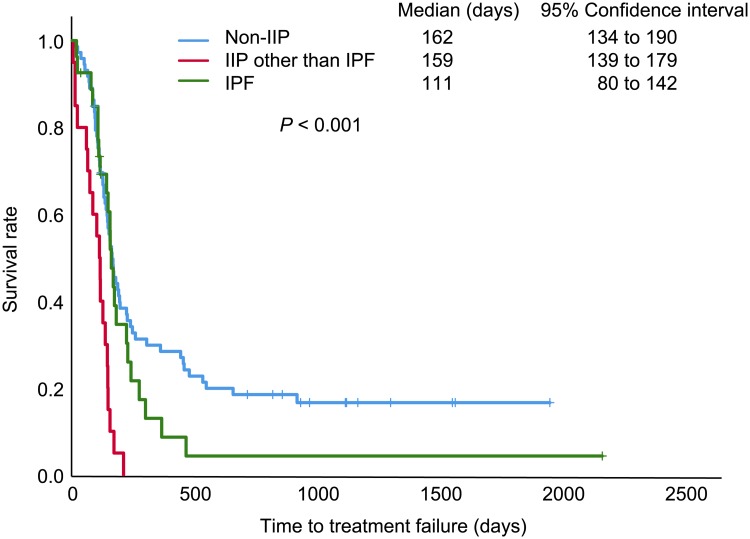
Kaplan-Meier curves for time to treatment failure of first-line chemotherapy. Using the log-rank test as univariate analysis, there were significant differences among the three patient groups: patients with small cell lung cancer with idiopathic pulmonary fibrosis (IPF), interstitial pneumonia (IIP) other than IPF, and non-IIP.

**Fig 3:**
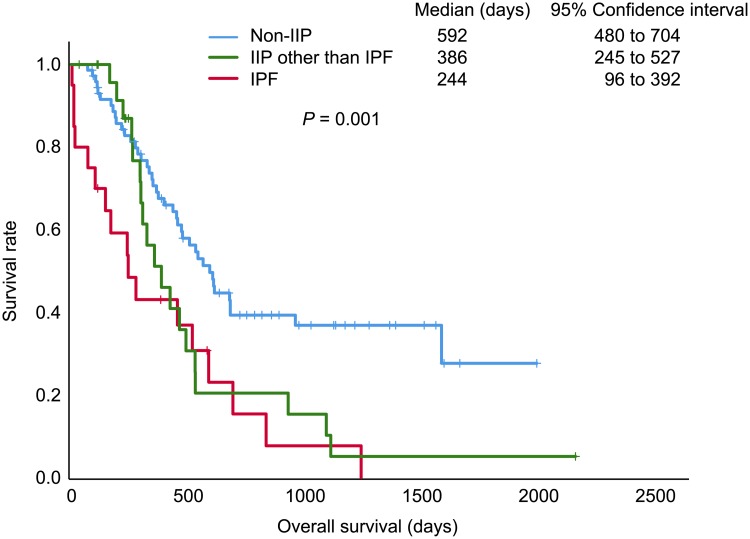
Kaplan-Meier curves for overall survival. Using the log-rank test as univariate analysis, there were significant differences among the three patient groups: patients with small cell lung cancer with idiopathic pulmonary fibrosis (IPF), interstitial pneumonia (IIP) other than IPF, and non-IIP.

In univariate analyses, ILD development/IIP exacerbation and comorbid IPF, but not other IIP, was significantly associated with time to treatment failure, and significant differences in overall survival were associated with multiple factors, including other IIP (Table 5, S1–S6 Figs). The Kaplan–Meier survival curves showed that patients with IPF had the shortest time to treatment failure (*P* < 0.001; Fig 2) and overall survival (*P* = 0.001; Fig 3), in comparisons of three groups. In the Cox proportional hazard model, time to treatment failure was shorter in patients with ILD development/IIP exacerbation (hazard ratio [HR]: 4.93, 95% confidence interval [CI]: 1.59–15.28, *P* = 0.006) and IPF (HR: 2.75, 95%CI: 1.20–6.30, *P* = 0.017), whereas other IIP was not significant (Table 5). Similarly, ILD development/IIP exacerbation (HR: 2.80, 95% CI: 1.06–7.44, *P* = 0.038) and IPF (HR: 3.17, 95% CI: 1.38–7.27, *P* = 0.006) were independently associated with worse overall survival, whereas other IIP showed no significance (Table 5).

**Table 5:** Univariate and multivariate analysis of time to treatment failure and overall survival.

	Time to treatment failure				Overall survival			
	Univariate analysis (log-rank test)	Multivariate analysis (Cox proportional hazard model)			Univariate analysis (log-rank test)	Multivariate analysis (Cox proportional hazard model)		
	*P*-value	*P* -value	Hazard ratio	95% CI	*P*-value	*P*-value	Hazard ratio	95% CI
**IPF**	0.001 >	0.017	2.75	1.20–6.30	0.003	0.006	3.17	1.38–7.27
**Other IIP**	0.723	0.263	0.71	0.39–1.29	0.104	0.940	1.03	0.50–2.11
**ILD development or IIP Exacerbation**	0.001 >	0.006	4.93	1.59–15.28	0.001 >	0.038	2.80	1.06–7.44
**Age (≥ 70 yo vs < 70 yo)**	0.061	0.621	0.89	0.55–1.43	0.011	0.996	1.00	0.56–1.79
**Sex**	0.814	0.174	0.68	0.38–1.19	0.572	0.167	0.59	0.28–1.25
**Smoking status (≥ 20 pack-year vs < 20 pack-year)**	0.614	0.757	1.09	0.65–1.83	0.894	0.666	0.87	0.46–1.64
**ECOG performance status (0–1 vs 2–3)**	0.011	0.021	1.37	1.05–1.78	0.001 >	0.015	1.57	1.09–2.26
**Stage (Limited stage vs Extensive stage)**	0.001 >	0.019	2.19	1.14–4.22	0.001 >	0.001 >	7.30	3.13–17.02
**1st-line chemotherapy regimen (cisplatin-based regimen vs other regimens)**	0.002	0.166	1.71	0.80–3.64	0.001 >	0.199	1.85	0.72–4.74
**Treatment courses of 1st-line chemotherapy (1–3 courses vs 4–6 courses)**	0.001 >	0.004	0.34	0.16–0.71	0.001 >	0.385	0.68	0.29–1.62
**Objective response of 1st-line chemotherapy (CR+PR vs SD+PD)**	0.001 >	0.001 >	0.20	0.10–0.42	0.001	0.604	0.82	0.39–1.74
**2nd-line treatment (Yes vs No)**	0.024	0.003	2.23	1.31–3.88	0.284	0.002	0.35	0.18–0.69
**Thoracic radiation therapy (Yes vs No)**	0.001 >	0.075	0.52	0.26–1.07	0.001	0.452	0.75	0.35–1.60
**Surgical intervention (Yes vs No)**	0.001 >	0.001 >	0.14	0.05–0.39	0.001 >	0.019	0.20	0.05–0.77

CI, confidence interval; IPF, idiopathic pulmonary fibrosis; Other IIP, idiopathic interstitial pneumonia other than idiopathic pulmonary fibrosis;

ILD, interstitial lung disease; IIP, idiopathic interstitial pneumonia; ECOG, eastern cooperative oncology group; CR, complete response; PR, partial response;

SD, stable disease; PD, progressive disease.

## Discussion

The purpose of this study was to investigate and compare clinical courses and prognoses between patients with comorbid SCLC with IPF, other IIP, and non-IIP. In the study, the incidence of chemotherapy-induced ILD development/IIP exacerbation in patients with IPF was significantly higher than that in other patient groups. This frequency was higher than that reported by Kenmotsu et al [17]. The incidence of 8.3% in patients with SCLC with IIP consisting of IPF and other IIP in this study also occurred within the range of 5.9–28.6% based on the previous reports [14, 15]. Along with the result from the multivariate logistic regression analysis for ILD development/IIP exacerbation, the high risk of ILD development/IIP exacerbation was also confirmed in patients with SCLC with IPF compared with those with other IIP and those without IIP.

Of the three patient groups that also showed significant differences in time to treatment failure and overall survival, the IPF group had significantly worse time to treatment failure and overall survival in both univariate and multivariate analyses, unlike the other IIP group that failed to show the difference in a multivariate analysis. These results indicate that IPF of IIP may be a negative prognostic factor for time to treatment failure and overall survival, independent of ILD development/IIP exacerbation, suggesting distinct natural history and clinical course in the IPF group compared with other patient groups.

Although IPF was identified as a negative prognostic factor and the high risk of ILD development/IIP exacerbation in patients with SCLC, the present study has some limitations. First, this retrospective study has differences among the numbers of patients in the three groups including a small number of SCLC patients with IPF, which may lead to an undefined potential bias. However, recent large-scale studies of comorbid lung cancer and IPF also comprised a small number of SCLC patients, and studies of only SCLC patients with IPF were conducted at a small scale: all analyses were based on data from less than 12 patients [24–28]. The results of this study were broadly consistent with those of previous studies, and furthermore, comparisons of the groups with IPF, other IIP, and non-IIP newly reached the result that comorbid IPF was an independent prognostic factor in the present study. Evidence from these small-scale studies may also need to be accumulated, although a large-scale study is obviously essential. Second, there may also be a potential bias of treatment between patients with and without IIP because some anti-tumor drugs, including irinotecan and amrubicin are contraindicated for patients with ILD and thoracic irradiation basically circumvents their high risk of radiation-induced ILD development/IIP exacerbation. In this study, two of 10 patients with ILD development/IIP exacerbation underwent concurrent chemoradiation therapy. However, ILD development/IIP exacerbation in these two patients occurred more than two months after the completion of thoracic irradiation, when treated with chemotherapy alone. Furthermore, multivariate analyses were also used to adjust for potential confounding factors involved in these limitations. Finally, ILD development/IIP exacerbation was diagnosed and classified based on the onset during chemotherapy and CT images in the present study. Other etiologies presenting with similar diffuse parenchymal image patterns, such as *Pneumocystis jirovecii* pneumonia, viral pneumonia, and carcinomatous lymphangiosis have not been pathologically excluded. However, test for β-D glucan levels, polymerase chain reaction assay for *Pneumocystis jirovecii* pneumonia, and tests for virus antibodies as a non-invasive assessment showed negative results. As for lymphangiosis, we confirmed no apparent progression of SCLC lesions at the time of onset. Furthermore, antibiotics were empirically initiated, although the results in sputum culture tests were negative.

## Conclusion

Comparisons among the three groups of SCLC patients without IIP, with IPF, and with other IIP, revealed that patients with IPF were at risk of decreased time to treatment failure and overall survival and chemotherapy-induced ILD development/IIP exacerbation. Comorbid IPF was significantly associated with ILD development/IIP exacerbation and was an independent, negative prognostic indicator. The findings of the present study suggest that an optimal therapeutic strategy should be individually provided to prevent the risk of ILD development/IIP exacerbation, particularly in patients with IPF. Currently, SCLC patients with IPF have limited therapeutic options other than a chemotherapeutic regimen combining platinum and etoposide. Given previous reports of the association between the timing of the initiation of corticosteroid treatment and prognosis in patients with exacerbated IPF, early diagnosis and intervention for chemotherapy-induced IIP exacerbation will be beneficial for SCLC patients with IPF, who need close monitoring for its onset [29, 30]. A large-scaled study at multiple institutes will be further warranted to evaluate the findings obtained from this small-scaled study.

## Supporting information

S1 Fig Kaplan-Meier curves for time to treatment failure of first-line chemotherapy. Using the log-rank test as univariate analysis, patients with small cell lung cancer with idiopathic pulmonary fibrosis (IPF) had shorter time to treatment failure than those without IPF.
(EPS)

S2 Fig Kaplan-Meier curves for time to treatment failure of first-line chemotherapy. Using the log-rank test as univariate analysis, there were no statistically significant differences in time to treatment failure between patients with small cell lung cancer with idiopathic interstitial pneumonia (IIP) other than idiopathic pulmonary fibrosis (IPF) and those without IIP other than IPF.
(EPS)

S3 Fig Kaplan-Meier curves for time to treatment failure of first-line chemotherapy. Using the log-rank test as univariate analysis, patients with small cell lung cancer who had development of interstitial lung disease or exacerbation of idiopathic interstitial pneumonia had shorter time to treatment failure than those who did not.
(EPS)

S4 Fig Kaplan-Meier curves for overall survival. Using the log-rank test as univariate analysis, patients with small cell lung cancer with idiopathic pulmonary fibrosis (IPF) had shorter overall survival than those without IPF.
(EPS)

S5 Fig Kaplan-Meier curves for overall survival. Using the log-rank test as univariate analysis, there were no statistically significant differences in overall survival between patients with small cell lung cancer with idiopathic interstitial pneumonia (IIP) other than idiopathic pulmonary fibrosis (IPF) and those without IIP other than IPF.
(EPS)

S6 Fig Kaplan-Meier curves for overall survival. Using the log-rank test as univariate analysis, patients who had development of interstitial lung disease or exacerbation of idiopathic interstitial pneumonia had shorter overall survival than those who did not.
(EPS)
